# Spike Protein Antibodies Mediate the Apparent Correlation between SARS-CoV-2 Nucleocapsid Antibodies and Neutralization Test Results

**DOI:** 10.1128/spectrum.00218-21

**Published:** 2021-06-16

**Authors:** Thomas Perkmann, Thomas Koller, Nicole Perkmann-Nagele, Miriam Klausberger, Mark Duerkop, Barbara Holzer, Boris Hartmann, Patrick Mucher, Astrid Radakovics, Maria Ozsvar-Kozma, Oswald F. Wagner, Christoph J. Binder, Helmuth Haslacher

**Affiliations:** a Department of Laboratory Medicine, Medical University of Vienna, Vienna, Austria; b Institute of Molecular Biotechnology, Department of Biotechnology, University of Natural Resources and Life Sciences (BOKU) Vienna, Vienna, Austria; c Institute of Bioprocess Science and Engineering, Department of Biotechnology, University of Natural Resources and Life Sciences (BOKU) Vienna, Vienna, Austria; d Department for Animal Health, Austrian Agency for Health and Food Safety (AGES), Moedling, Austria; University of Arizona/Banner Health

**Keywords:** SARS-CoV-2, neutralizing antibodies, serology

## TO THE EDITOR

Although SARS-CoV-2 induces a variety of different antibodies, not all of them act as neutralizing antibodies. Whereas antibodies against the viral spike protein, which contains the receptor-binding domain (RBD), are known to have neutralizing properties (1–4), some authors have also shown that antibodies against the nucleocapsid protein (NC) correlate with neutralization activity (5). However, these structures are not present on the viral surface (2, 6, 7). Consequently, it has been hypothesized that these are mere spurious correlations mediated by the cooccurrence of antibodies to RBD (2, 8).

To prove this hypothesis, we measured levels of antibodies binding to the NC and the S antigens in 64 individuals with previous SARS-CoV-2 infection (9) by various CE-labeled immunoassays (Roche total antibody NC electrochemiluminescence immunoassay [ECLIA], Abbott IgG NC chemiluminescent microparticle assay [CMIA], Technozym RBD IgG enzyme-linked immunosorbent assay [ELISA], and DiaSorin S1/S2 IgG chemiluminescence assay [CLIA]). The functional ability of sera to neutralize SARS-CoV-2 was tested using a classical 50% tissue culture infectious dose assay (neutralization tests [NT]) as previously described (10). Recruiting procedures, preanalytical workflows, and analytical methods were reported earlier (9, 11, 12).

**FIG 1:**
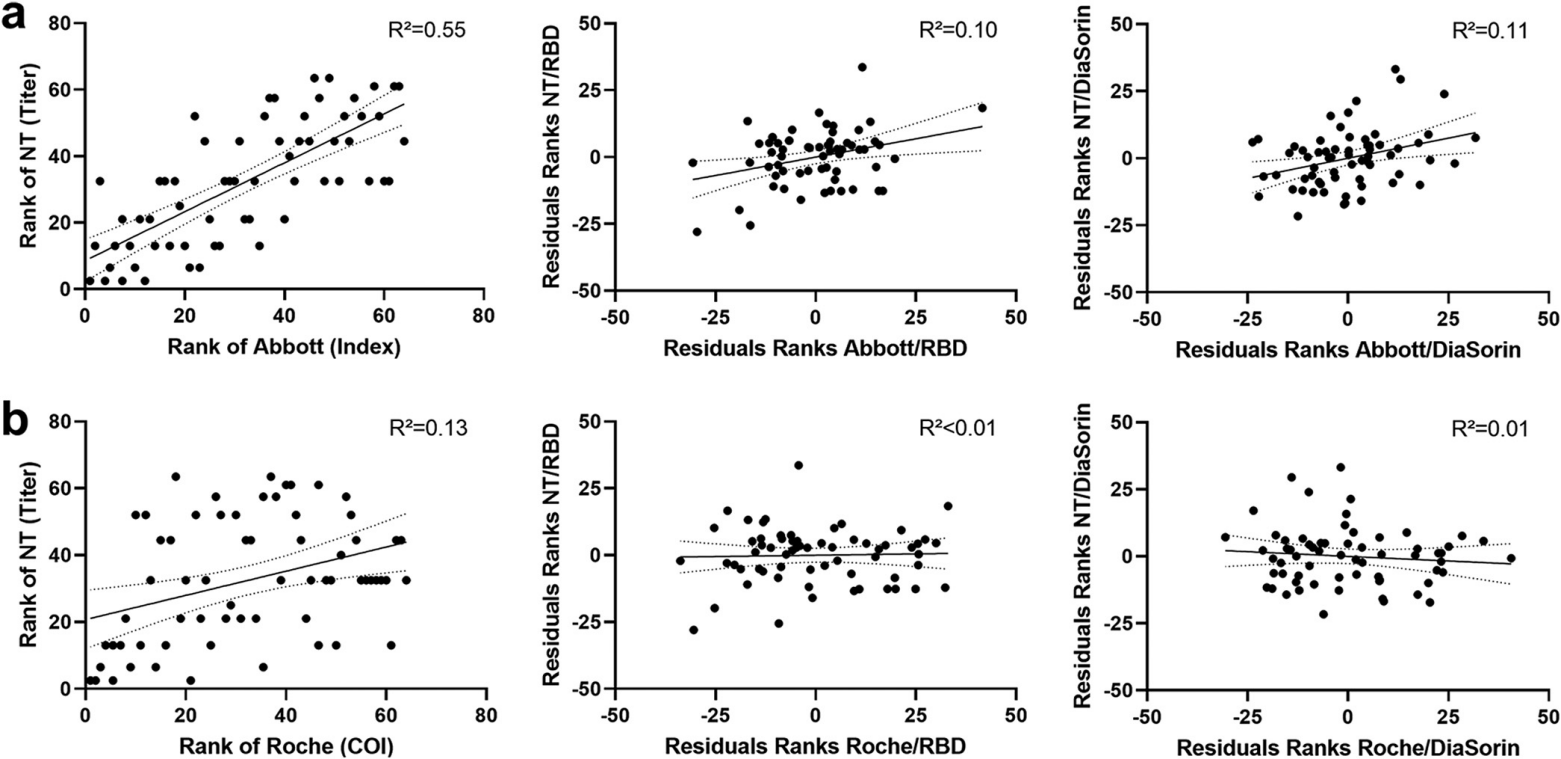
Rank correlations between titers of viral neutralization tests (NT) and nucleocapsid antibody concentrations measured by the (a) Abbott CMIA (IgG) or the (b) Roche ECLIA (IgG, IgM, and IgA). The figures in the second column show the changes in rank correlations by keeping the RBD (Technozym) or S1/S2 (DiaSorin) IgG antibody concentration constant. *, *P* < 0.05; **, *P* < 0.01; ****, *P* < 0.0001; *R*^2^, coefficient of determination.

Both tested assays assessing antibodies against the NC correlated significantly with NT titers: Abbott, ρ = 0.742, *P* = 2.2 × 10^−12^; Roche, ρ = 0.365, *P* = 0.003. However, if the rank correlations were controlled for RBD antibody presence, correlation coefficients dropped to ρ = 0.318 (*P* = 0.011; Abbott) and ρ = 0.032 (*P* = 0.806; Roche). The same holds if antibodies against an S1/S2 combination antigen were kept constant: ρ = 0.329 (*P* = 0.008; Abbott), ρ = −0.101 (*P* = 0.430; Roche). When RBD or S1/S2 antibody concentrations were not considered, up to 55% of NT titer variability could be explained by NC antibody concentrations (Abbott) (Fig. 1).

**FIG 2:**
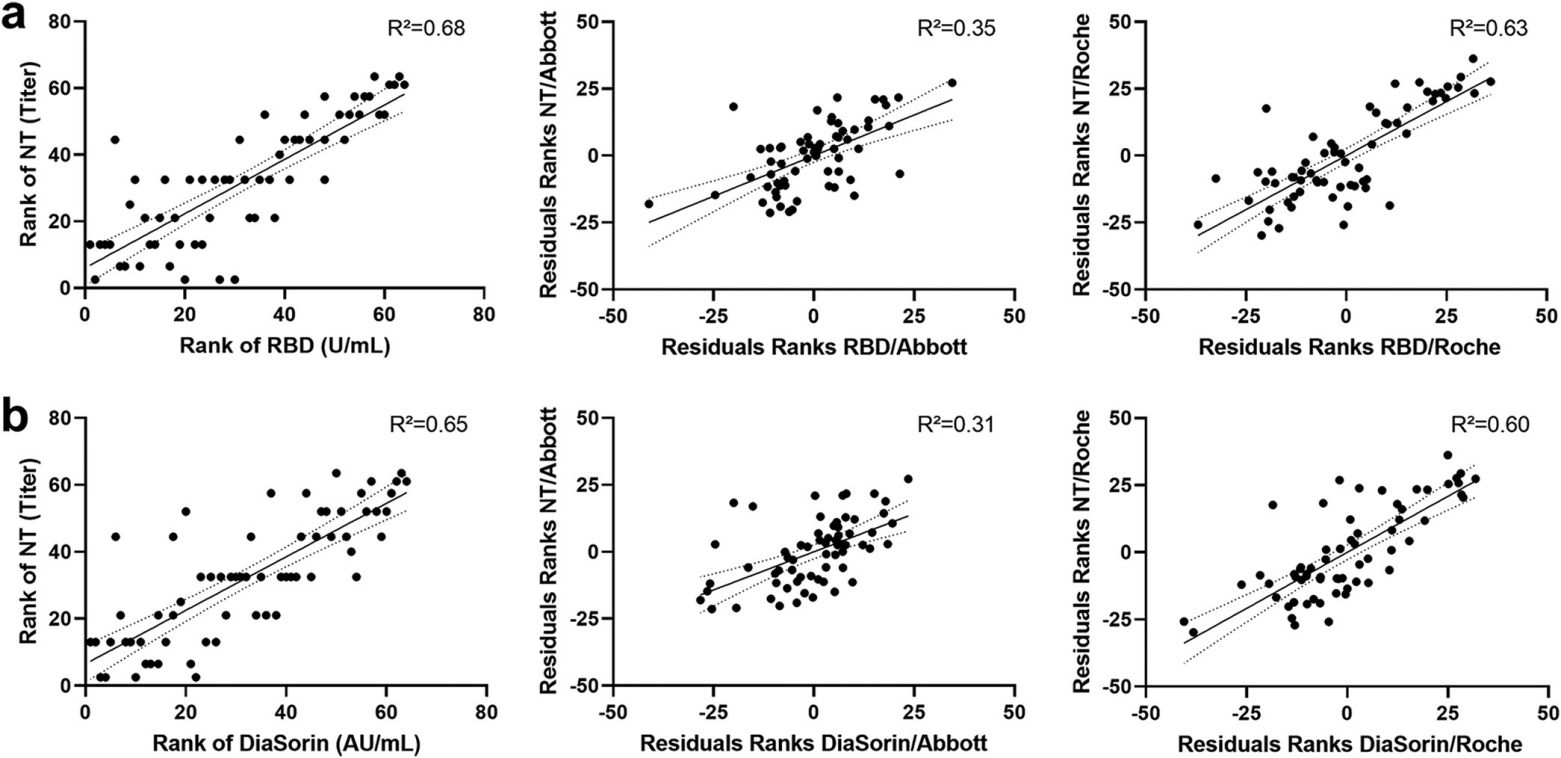
Rank correlations between titers of viral neutralization tests (NT) and RBD antibody concentrations quantified by the Technozym RBD ELISA (a) or S1/S2 antibodies (b). The figures in the second and third columns present rank correlations between NT and RBD with anti-nucleocapsid antibody concentrations kept constant in two different assays (Abbott, Roche). *R*^2^, coefficient of determination.

In a last step, we aimed to assess whether the correlation between RBD antibody concentrations and NT titers could be attenuated to the same extent by keeping NC antibody concentrations constant. Although controlling for Abbott NC IgG levels affected the coefficients of determination, the remaining *R*^2^ was still 0.31 for S1/S2 and 0.35 for RBD antibodies (Fig. 2).

Our data suggest that the relationship between NC antibody levels and NT titers is only apparent and is mostly mediated by the concomitant presence of RBD or S1/S2 antibodies. Specific S antibody concentrations and NT titers remain correlated after keeping NC antibody levels constant. In contrast, the correlation between NC antibody levels and NT titers nearly vanished for most of the assessed test systems after keeping RBD or S1/S2 antibody levels constant. This was especially true for Roche NC assay measured total NC antibody levels, whereas the IgG-based assays appeared to correlate better with NT titers. The remaining weak correlation between the Abbott test results and the NT titers, while keeping RBD or S1/S2 IgG antibody concentrations constant, suggests that a third mediator variable is present.

To prevent falsely implied causal relationships between SARS-CoV-2-specific NC antibodies and neutralizing activity, all correlation analyses of non-spike-associated antibody assays and neutralization assays should include partial correlation analyses to exclude a possible mediator effect of spike-associated antibodies. In addition, clinicians should be aware of the noncausality of correlations between antinucleocapsid antibodies and neutralization test titers when interpreting laboratory findings.
